# Transitions between versions of the International Classification of Diseases and chronic disease prevalence estimates from administrative health data: a population-based study

**DOI:** 10.1186/s12889-022-13118-8

**Published:** 2022-04-09

**Authors:** Ridwan A. Sanusi, Lin Yan, Amani F. Hamad, Olawale F. Ayilara, Viktoriya Vasylkiv, Mohammad Jafari Jozani, Shantanu Banerji, Joseph Delaney, Pingzhao Hu, Elizabeth Wall-Wieler, Lisa M. Lix

**Affiliations:** 1 Department of Community Health Sciences, University of Manitoba, Winnipeg, MB R3E0T6 Canada; 2 Department of Statistics, University of Manitoba, Winnipeg, MB R3T2N2 Canada; 3 CancerCare Manitoba Research Institute, Rady Faculty of Health Sciences, University of Manitoba, Winnipeg, MB R3EOV9 Canada; 4 College of Pharmacy, University of Manitoba, Winnipeg, MB R3E0T5 Canada; 5 Department of Epidemiology, University of Washington, Seattle, WA 98195 USA; 6 Department of Biochemistry and Medical Genetics, University of Manitoba, Winnipeg, MB R3E0J9 Canada

**Keywords:** Diagnosis codes, Hotelling’s T^2^, Multivariate control chart, Negative binomial, Trend analysis

## Abstract

**Background:**

Diagnosis codes in administrative health data are routinely used to monitor trends in disease prevalence and incidence. The International Classification of Diseases (ICD), which is used to record these diagnoses, have been updated multiple times to reflect advances in health and medical research. Our objective was to examine the impact of transitions between ICD versions on the prevalence of chronic health conditions estimated from administrative health data.

**Methods:**

Study data (i.e., physician billing claims, hospital records) were from the province of Manitoba, Canada, which has a universal healthcare system. ICDA-8 (with adaptations), ICD-9-CM (clinical modification), and ICD-10-CA (Canadian adaptation; hospital records only) codes are captured in the data. Annual study cohorts included all individuals 18 + years of age for 45 years from 1974 to 2018. Negative binomial regression was used to estimate annual age- and sex-adjusted prevalence and model parameters (i.e., slopes and intercepts) for 16 chronic health conditions. Statistical control charts were used to assess the impact of changes in ICD version on model parameter estimates. Hotelling’s T^2^ statistic was used to combine the parameter estimates and provide an out-of-control signal when its value was above a pre-specified control limit.

**Results:**

The annual cohort sizes ranged from 360,341 to 824,816. Hypertension and skin cancer were among the most and least diagnosed health conditions, respectively; their prevalence per 1,000 population increased from 40.5 to 223.6 and from 0.3 to 2.1, respectively, within the study period. The average annual rate of change in prevalence ranged from -1.6% (95% confidence interval [CI]: -1.8, -1.4) for acute myocardial infarction to 14.6% (95% CI: 13.9, 15.2) for hypertension. The control chart indicated out-of-control observations when transitioning from ICDA-8 to ICD-9-CM for 75% of the investigated chronic health conditions but no out-of-control observations when transitioning from ICD-9-CM to ICD-10-CA.

**Conclusions:**

The prevalence of most of the investigated chronic health conditions changed significantly in the transition from ICDA-8 to ICD-9-CM. These results point to the importance of considering changes in ICD coding as a factor that may influence the interpretation of trend estimates for chronic health conditions derived from administrative health data.

**Supplementary Information:**

The online version contains supplementary material available at 10.1186/s12889-022-13118-8.

## Introduction

International Classification of Diseases (ICD) codes were developed by the World Health Organization (WHO) as the worldwide standard for classifying the causes of injury and death [1]. These codes are captured in administrative health data, such as hospital records. ICD codes have been updated multiple times to reflect advances in health and medical science, and these changes are reflected in administrative health data. For example, US administrative health billing data changed from the 9^th^ revision Clinical Modification (ICD-9-CM) to the 10^th^ revision (ICD-10-CM) in 2015 [2]. Several countries, including Australia (ICD-10-AM), Canada (ICD-10-CA), Germany (ICD-10-GM), Korea (ICD-10-KM), and Thailand (ICD-10-TM), developed ICD-10 modifications to address country-specific needs [3].

In Canada, three ICD versions are captured in many administrative health databases: 8^th^ revision (ICD-8), 9^th^ revision (ICD-9), and 10^th^ revision (ICD-10), which were introduced by the WHO in 1965, 1975, and 1993, respectively [4–6]. Each ICD version has a greater number of codes, resulting in new diseases being added, and other diseases being recategorized, removed, or combined [1]. Though the increasing level of detail with each update of the ICD system is essential for diagnostic and administrative purposes, codes in one ICD version may not map (i.e., correspond) exactly to codes in another ICD version [7]. This introduces challenges in using ICD codes to track trends in disease prevalence. A change in the trend may be associated with changes in coding standards rather than a change in the true disease prevalence.

Heslin and Barrett [8] observed an upward shift in the number of diagnosed cases of alcohol abuse, alcohol-induced mental disorders, and intoxication after ICD-10-CM was introduced as compared to when ICD-9-CM was used in the US. The study used 2-sided t-statistics to test for a difference in the average quarterly counts of inpatient stays between ICD periods. Slavova et al. [2] used segmented regression to model injury hospitalizations to evaluate the effect of transitioning from ICD-9-CM to ICD-10-CM in the US; they reported a significant change in the slope estimate after the transition in 2015. The effects of transitions to a new ICD version help researchers to know where to expect significant and sustained changes in trends between one ICD version and another [9].

A control chart, an efficient statistical tool to monitor and signal changes in a process over time [10], can also be used to investigate the trend pattern before and after transitions to a different ICD version. The control chart was first introduced in manufacturing for monitoring product estimates, such as the number of defects [11]. The control chart has been used in population health and health services research and surveillance to monitor trend estimates, such as outcomes of pneumonia [12], proportion of live births by caesarean section [13], ratio of nurse attendance to ward workload [14], mortality rates [15], morbidity rates of patients after undergoing coronary artery bypass graft surgery [16], measurement error in vital signs [17﻿], and patient dissatisfaction with hospitals [18]. As well, Hanslik et al. [19] implemented the control chart as an epidemiological tool to test for a significant increase in the average number of cases of communicable, environmental and societal diseases relating to mass gatherings. Coory et al. [20] used the control chart to monitor clinical indicators in administrative health data. Other control chart applications include monitoring infection rates and lengths of hospital stays [21]. Control charts could also be used to investigate changes in disease trends amongst ICD versions.

This study applied control charts for the surveillance of chronic health conditions over time. Our objective was to examine the impact of transitions between ICD versions on the prevalence of chronic health conditions estimated from administrative health data.

## Methods

### Data sources

Study data were from the province of Manitoba, Canada, which has a population of approximately 1.3 million according to the 2016 Statistics Canada Census [22]. Manitoba has a universal healthcare system for publicly-funded services, which include hospitalizations, prescription drug dispensations, and outpatient physician visits. More than 99% of the population is eligible to receive health insurance coverage. Details of who is captured and excluded from the Manitoba Health Insurance Registry is provided by Hamm et al. [23]*.*

The Manitoba Population Research Data Repository housed at the Manitoba Centre for Health Policy, University of Manitoba, has a variety of administrative health databases that contain ICD codes and can be linked via a unique, anonymized personal health identifier. The specific databases used in this study were the Medical Services database, the Hospital Discharge Abstracts Database (DAD), and the Manitoba Health Insurance Registry (Table 1). The Medical Services database consists of physician billing claims, which are forwarded to the ministry of health for reimbursement of fee-for-service physicians. Each record includes the date of service and a single ICD code that corresponds to the reason for the physician visit. The diagnoses were recorded using the 8^th^ revision of ICD with adaptations (ICDA-8) from 1970 until 1979, when ICD-9-CM was adopted. Physician billing claims capture visits to family physicians and specialists provided in outpatient (i.e., clinic) settings. This database covers more than 80 provider categories and multiple specialist fields [24]. The DAD contains hospital records for all acute care facilities in the province; it does not capture emergency department visits. Diagnosis codes in the DAD were defined using ICDA-8 codes from 1970 to 1979. After this period, ICD-9-CM was used until 2004, when ICD-10-CA (ICD-10 with Canadian enhancement) was adopted. The Manitoba Health Insurance Registry contains records for all individuals eligible for health insurance coverage in the province [25]. It also captures the start and end dates of coverage and socio-demographic information. The study databases were used to define the annual study cohorts from 1974 to 2018, as well as to produce demographic characteristics and prevalence estimates of chronic health conditions. The accuracy and completeness of Manitoba’s administrative data have been demonstrated in multiple studies and tools for data quality assessment have been developed and are routinely applied to the data [26–29].

**Table 1:** Characteristics of the study data

Years of Study Data	Data Sources	ICD Version
1974–1979	Medical Services Database (Physician billing claims)	ICDA-8
	Hospital Discharge Abstracts Database (Hospital records)	ICDA-8
	Manitoba Health Insurance Registry	N/A
1980–2004	Medical Services Database (Physician billing claim)	ICD-9-CM
	Hospital Discharge Abstracts Database (Hospital records)	ICD-9-CM
	Manitoba Health Insurance Registry	N/A
2005–2018	Medical Services Database (Physician billing claim)	ICD-9-CM
	Hospital Discharge Abstracts Database (Hospital records)	ICD-10-CA
	Manitoba Health Insurance Registry	N/A

*ICD* International Classification of Diseases, *N/A* No diagnoses are recorded in Registry data

### Study cohorts

The study cohorts were formed by including all individuals residing in Manitoba that were 18 years of age or older in each year between 1974 and 2018. We categorised age into the following groups: 18–29, 30–39, 40–44, 45–49, 50–54, 55–59, 60–64, 65–69, and 70 years and above. Individuals with less than three years of continuous health coverage were excluded.

### Identifying chronic health conditions in administrative health data

We selected 16 chronic health conditions for this study that include both physical health and mental health. The chronic health conditions include rare and common conditions, those that affect a range of body systems; and conditions that can adversely affect health-related quality of life. Thus, the selected conditions provide a good representation of the variety of health conditions captured by the ICD system. The chronic health conditions were selected from multiple chronic health conditions derived using Clinical Classification Software (CCS) [7]. The CCS, developed by the Agency for Healthcare Research and Quality in the US, has been adopted in many studies to provide clinically meaningful and interpretable statistics such as disease prevalence, frequencies, and medical expenditures [30, 31]. Crosswalks of diagnoses codes to CCS categories are provided by Hamad et al. [7]. The selected chronic health conditions include mood and anxiety disorders, menstrual disorders, hypertension, osteoarthritis, anemia, diabetes, asthma, acute myocardial infarction, heart valve disorders, acute cerebrovascular disease, cataracts, breast cancer, colon cancer, lung and respiratory cancers, prostate cancer, and skin cancer.

### Statistical analyses

﻿ The analyses were conducted for each year in the 45-year study period (1974 to 2018). Descriptive statistics, including means, standard deviations, and percentages, were used to describe the study cohorts’ demographic characteristics. We fit negative binomial regression models $(M1)$, a generalization of Poisson regression models $(M2)$ that loosen the restrictive assumption that the variance is equal to mean, to counts of the number of cases of each chronic health condition in each age group and sex strata for the ${j}^{th}$ year (*j* = 1,…, *m; m* = 45). Model goodness of fit was assessed using a likelihood ratio test with a nominal significance level of α = 0.01, to reduce the likelihood of a Type 1 error. We also compared the models’ ratio of residual deviance to degrees of freedom (df) [32] for both the negative binomial and Poisson regression models. The natural logarithm of the population size in each age group and sex strata was the model offset. Sex and age group were the model covariates. We estimated the annual age- and sex-adjusted prevalence and annual regression coefficients $\widehat{{{\varvec{\beta}}}_{\mathrm{j}}}=\left({b}_{kj}\right), k=0, 1, \dots ,(p-1)$, where $p=10$ is the number of estimated parameters. The regression model parameters for the ${j}^{th}$ year include the intercept (${b}_{0j}$) and the slopes $\left({b}_{1j},\dots ,{b}_{9j}\right)$ where the reference categories were female for sex and 40–44 years for age group.

We estimated the average annual rate of change, expressed as a percentage, in the age- and sex-adjusted prevalence for three time segments. In segment 1 (1974–1979) diagnosis codes were defined using ICDA-8 in both data sources (i.e., physician billing claims and hospital records). In segment 2 (1980–2004), diagnosis codes were defined using ICD-9-CM in both data sources. In segment 3 (2005–2018), diagnosis codes were defined using ICD-10-CA in hospital records, while in physician billing claims they were still defined using ICD-9-CM.

We used Hotelling’s T^2^ control chart [33] to monitor and signal changes in the regression model parameter estimates for each year of the study period. We hypothesized that stability of the model parameters is an indicator of stability in the prevalence estimates. Hotelling’s T^2^ statistic simultaneously monitors the regression model parameter estimates; an out-of-control signal occurs if the statistic is greater than a pre-specified control limit value [17]. This approach had been described previously [34–37]; Woodall et al. [35] noted that since the estimators of intercept and slope are dependent, it is reasonable to monitor them together.

We used the Durbin-Watson test statistic to detect the presence of autocorrelation (and to estimate the autocorrelation coefficient) between years [38], because the same individuals may be captured in prevalence estimates for subsequent years. Noorossana et al. [39] performed a simulation study to illustrate a significant decrease in control charts performance when autocorrelation is overlooked. To reduce the impact of autocorrelation, we used a U-statistic method (see U-Statistic Definition in Additional file 1), where the estimate for the ${j}^{th}$ year is adjusted for correlation in the preceding (i.e., *j* – 1) year [38, 40].

Hotelling’s T^2^ statistic [17, 41] for the *j*th year is defined as.

1 $${T^2}_{j}\;={({{\mathbf{U}}}_{j}-{{\varvec{\upmu}}}_{U})}^{'}{{\varvec{\Sigma}}}^{-1}_{U}({{\mathbf{U}}}_{j}-{{\varvec{\upmu}}}_{U})$$

and plotted against an upper control limit (UCL) of.

2 $$UCL\;=\;\frac{p(m-1)(m+1)}{m(m-p)}F_{\alpha,p,m-p}$$

and a lower control limit (LCL) of zero. In Eq. 1, ${\mathbf{U}}_{j}$ is the vector of adjusted regression model parameters that are assumed to be independently and normally distributed with mean ${{\varvec{\upmu}}}_{U}$ and covariance matrix ${{\varvec{\Sigma}}}_{U}$. In Eq. 2, *F* is the critical value of the *F* distribution with degrees of freedom *p* and *m*—*p*, and $\alpha$ is the nominal level of significance. The control limit was 19.8 when α = 0.01. The mean vector ${{\varvec{\upmu}}}_{U}$ and covariance matrix ${{\varvec{\Sigma}}}_{U}$ can be estimated from a reference sample (or a training dataset) [20, 42]. In the absence of a reference sample, the mean vector and covariance matrix can be estimated from ${\mathbf{U}}_{j}$ [20, 42] by finding the mean and covariance of ${\mathbf{U}}_{j}$ across all $j$. The test statistic is said to be an in-control observation when it is within the bounds of LCL and UCL, otherwise it is an out-of-control observation. The presence of at least one out-of-control observation in a transition period was used to decide if changes in the ICD version affected the prevalence estimate of a chronic health condition. A transition period was defined as $\pm 2$ years around a transition year, where the transition year was 1979 when transitioning from ICDA-8 to ICD-9-CM and the transition year was 2005 when transitioning from ICD-9-CM to ICD-10-CA. Thus, the first transition period was from 1977 to 1981 and the second transition period was from 2003 to 2007. In a sensitivity analysis, we defined a transition period as $\pm 1$ year around a transition year. Since the DAD was the only data source that contains diagnoses recorded using ICD-10-CA, we also conducted separate analyses for each of the data sources (i.e., in physician billing claims, we focused on the transition period from ICDA-8 to ICD-9-CM, while in hospital records, we focused on the transition periods from ICDA-8 to ICD-9-CM and ICD-9-CM to ICD-10-CA).

## Results

### Characteristics of the study cohorts

The demographic characteristics of the study cohorts are described in Table 2 for selected study years. The annual cohort sizes increased from 360,341 in 1974 to 824,816 in 2018, while the average age increased from 37.3 years to 48.0 years, reflecting the growth and ageing of the Manitoba population over time.

**Table 2:** Demographic characteristics of the study cohorts in selected study years

Characteristics	1974	1984	1994	2004	2018
*N*	360,341	554,894	656,290	726,005	824,816
*Age,* mean (SD)	37.3 (13.0)	38.5 (15.1)	42.0 (16.7)	45.2 (17.6)	48.0 (18.8)
*Female* (%)	49.0	50.0	50.8	51.4	51.2

*SD* Standard Deviation

### Goodness of fit tests for regression models

We compared the goodness of fit of $M1$ (negative binomial model) and $M2$ (Poisson model) for each year in the study period using a likelihood ratio test and rejected the null hypothesis (dispersion parameter is infinity) for all the chronic health conditions because the resulting p-values were less than $0.01$. This implies that the data was over-dispersed and $M1$ was a better fit to the data. Also, the ratios of residual deviance to df were small and close to one for $M1$, unlike for $M2$ where the ratios were high (see Figure S1, Additional file 1). This indicates that $M2$ did not account for the standard errors of the over-dispersed data.

### Age- and sex-adjusted prevalence

Table 3 presents the age- and sex-adjusted prevalence (per 1,000 population) and the average annual rate of change (%) in prevalence for the chronic health conditions during the three time segments in which different ICD versions were used in the administrative data. Mood and anxiety disorders were the most common health condition in segments 1 and 2 with prevalence estimates of 74.4 and 138.8 in 1974 and 2004, respectively, and hypertension was the most prevalent health condition in segment 3 with prevalence estimates of 142.6 and 223.6 in 2005 and 2018, respectively. Skin cancer was the least diagnosed health condition in all the segments, with prevalence estimates of 0.3 and 2.1 in 1974 and 2018, respectively.

**Table 3:** Prevalence and average annual rate of change (%) in prevalence for chronic health conditions

**Chronic Health Condition**	**Segment 1: 1974—1979**			**Segment 2: 1980—2004**			**Segment 3: 2005—2018**		
	**Prevalence**		**Average annual rate of change (95% CI)**	**Prevalence**		**Average annual rate of change (95% CI)**	**Prevalence**		**Average annual rate of change (95% CI)**
	1974	1979		1980	2004		2005	2018	
Mood and anxiety disorders	74.4	74.0	8.0 (7.1, 8.9)	84.8	138.8	4.5 (4.4, 4.5)	138.8	165.7	3.0 (2.9, 3.1)
Menstrual disorders	63.5	59.1	2.4 (2.0, 2.8)	51.2	34.8	1.9 (1.7, 2.1)	32.7	26.2	0.0 (-0.1, 0.1)
Hypertension	40.5	62.5	14.6 (13.9, 15.2)	64.5	141.1	2.9 (2.8, 3.0)	142.6	223.6	3.9 (3.8, 4.0)
Osteoarthritis	15.7	18.4	9.4 (8.5, 10.2)	16.5	47.1	5.1 (5.0, 5.2)	52.0	55.5	0.8 (0.6, 0.9)
Anemia	15.3	13.0	2.6 (0.7, 4.5)	12.6	22.7	3.5 (3.3, 3.7)	21.2	26.5	3.8 (3.4, 4.2)
Diabetes	11.4	14.9	10.8 (10.3, 11.4)	14.7	57.3	6.3 (6.2, 6.4)	60.1	96.8	4.4 (4.3, 4.5)
Asthma	8.2	9.4	8.3 (7.4, 9.3)	10.3	35.3	7.4 (7.3, 7.5)	34.8	47.6	5.5 (5.3, 5.6)
Acute myocardial infarction	3.0	2.9	3.5 (3.0, 4.1)	2.9	4.1	0.9 (0.7, 1.0)	4.1	3.0	-1.6 (-1.8, -1.4)
Heart valve disorders	2.4	3.5	13.6 (12.5, 14.7)	2.7	3.6	1.3 (1.1, 1.4)	3.4	4.8	2.6 (2.5, 2.7)
Acute cerebrovascular disease	1.9	2.4	9.6 (8.4, 10.9)	2.4	6.9	4.3 (4.2, 4.5)	7.1	7.4	1.5 (1.4, 1.7)
Cataracts	1.7	2.4	10.3 (9.5, 11.2)	2.7	24.4	6.4 (6.3, 6.6)	24.5	25.9	1.5 (1.3, 1.6)
Breast cancer	1.3	1.8	10.0 (9.3, 10.7)	1.8	6.7	5.3 (5.1, 5.5)	6.9	6.0	-0.8 (-0.9, -0.7)
Colon cancer	0.5	0.6	8.8 (7.4, 10.2)	0.7	2.8	6.5 (6.4, 6.6)	2.8	2.9	1.5 (1.4, 1.7)
Lung & respiratory cancers	0.4	0.6	11.7 (10.3, 13.1)	0.6	1.9	4.3 (4.2, 4.5)	2.0	2.3	1.1 (0.9, 1.2)
Prostate cancer	0.3	0.5	9.3 (7.5, 11.0)	0.5	5.1	5.8 (5.0, 7.0)	5.1	6.2	2.4 (2.3, 2.6)
Skin cancer	0.3	0.3	10.5 (8.9, 12.1)	0.3	0.9	5.4 (5.2, 5.5)	0.9	2.1	5.9 (5.6, 6.1)

*Note:* Age- and sex-adjusted prevalence per 1,000 population

The highest average annual rate of change (%) in prevalence was recorded for hypertension in segment 1 (14.6; 95% confidence interval [CI]: 13.9, 15.2), asthma in segment 2 (7.4; 95% CI: 7.3, 7.5), and skin cancer in segment 3 (5.9; 95% CI: 5.6, 6.1). The lowest average annual rate of change in prevalence was recorded for menstrual disorders in segment 1 (2.4; 95% CI: 2.0, 2.8); acute myocardial infarction in segment 2 (0.9; 95% CI: 0.7, 1.0); and acute myocardial infarction in segment 3 (-1.6; 95% CI: -1.8, -1.4). Acute myocardial infarction and breast cancer showed a significant decline in prevalence during segment 3, while other health conditions showed an increase (Table 3).

### Control chart results

**Fig. 1:**
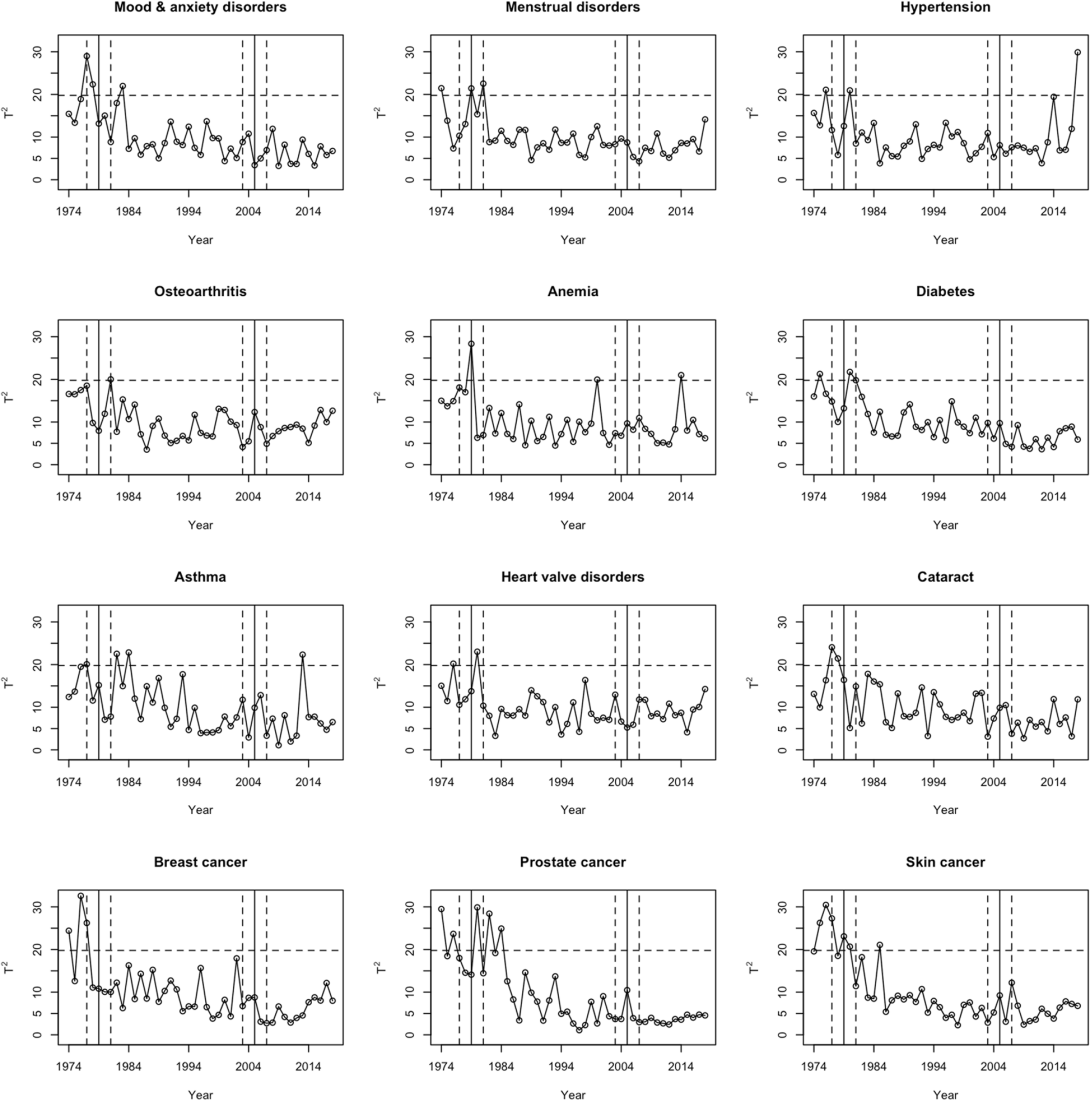
Chronic health conditions with significant changes in regression model parameter estimates within transition periods. Legend: Data sources are physician billing claims and hospital records; A transition period was defined as $\pm 2$ years around the transition year of 1979 for the transition from ICDA-8 to ICD-9-CM and around the transition year of 2005 for the transition from ICD-9-CM to ICD-10-CA; Horizontal dashed line represents the upper control limit of 19.8; Vertical solid lines represent transition years (1979 and 2005); Vertical dashed lines represent the beginning and end of the transition periods (1977—1981 and 2003—2007)

Figure 1 displays the chronic health conditions with significant changes in regression model parameter estimates when transitioning from one ICD version to another. Within the first transition period, there was at least one out-of-control observation (i.e., at least one Hotelling’s T^2^ statistic greater than the UCL) for each of the investigated chronic health conditions. The maximum Hotelling’s T^2^ statistics for mood and anxiety disorders, menstrual disorders, and hypertension in the first transition period were 29.0, 22.5, and 20.9, respectively, which are greater than the UCL (Table 4). However, in the second transition period, there were no out-of-control observations for any chronic health conditions.

**Table 4:** Summary of Hotelling’s T^2^ statistics for chronic health conditions in the transition periods

Chronic Health Condition	Transition period from ICDA-8 to ICD-9-CM		Transition period from ICD-9-CM to ICD-10-CA	
	**Max T**^**2**^	**Average T**^**2**^	**Max T**^**2**^	**Average T**^**2**^
Mood and anxiety disorders	**29.0**	17.7	10.8	7.0
Menstrual disorders	**22.5**	16.5	9.7	7.3
Hypertension	**20.9**	11.9	10.9	7.6
Osteoarthritis	**20.0**	13.6	12.3	7.1
Anemia	**28.4**	15.3	11.0	8.6
Diabetes	**21.7**	15.9	9.8	6.9
Asthma	**20.1**	12.3	12.8	8.1
Acute myocardial infarction	16.0	10.2	10.4	9.5
Heart valve disorders	**23.0**	13.9	12.9	8.5
Acute cerebrovascular disease	19.5	14.5	12.8	8.0
Cataracts	**24.1**	16.4	10.5	6.9
Breast cancer	**26.2**	13.7	8.8	6.0
Colon cancer	17.2	12.7	9.5	7.9
Lung & respiratory cancers	17.3	12.0	13.7	10.2
Prostate cancer	**29.9**	18.2	10.5	5.0
Skin cancer	**27.3**	20.2	12.2	6.5

Data sources are physician billing claims and hospital records; Boldface font indicates a test statistic value greater than the upper control limit of 19.8; The transition period was defined as $\pm 2$ years around the transition year of 1979 for the transition from ICDA-8 to ICD-9-CM, and around the transition year of 2005 for the transition from ICD-9-CM to ICD-10-CA

**Fig. 2:**
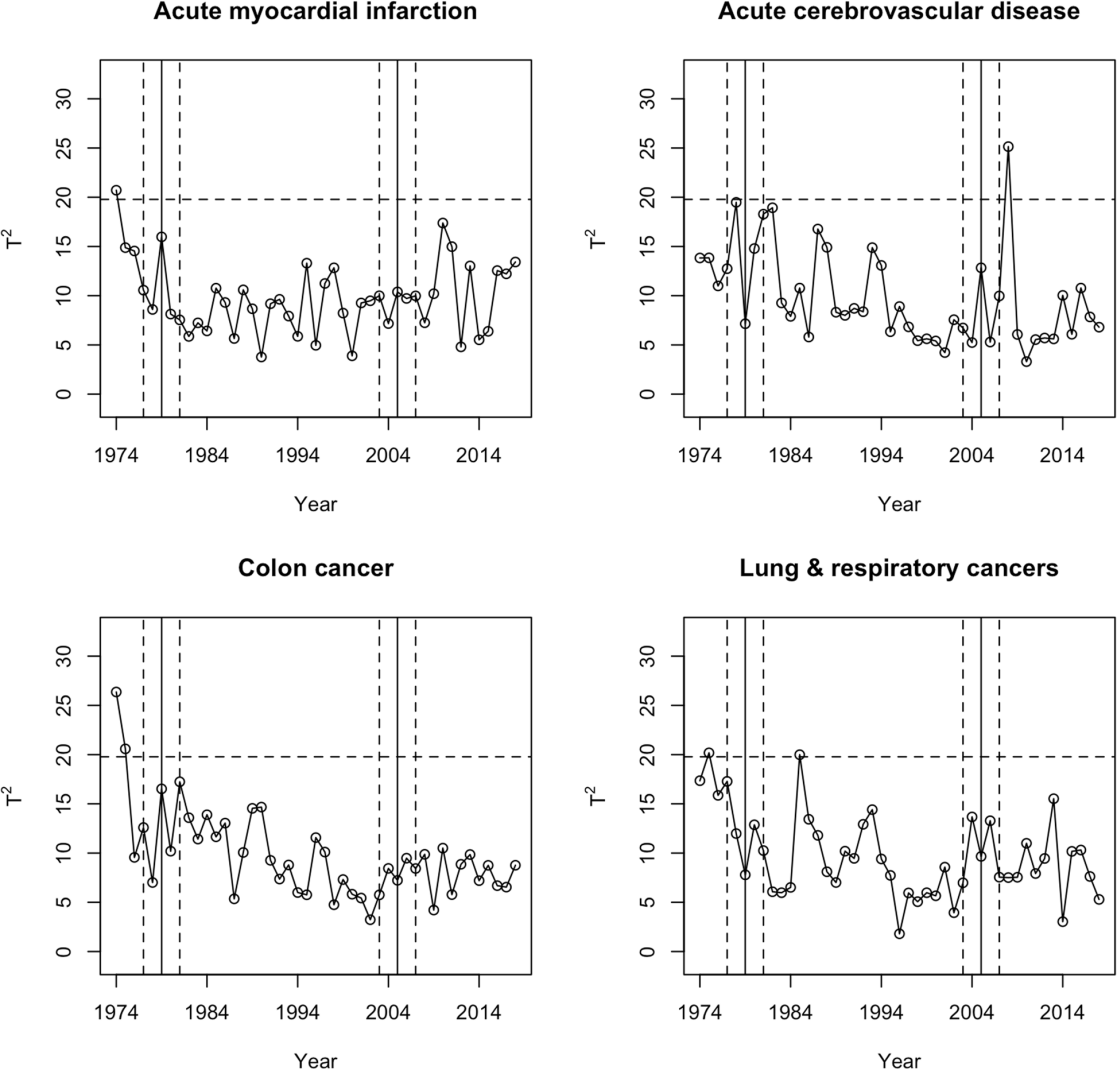
Chronic health conditions with no significant changes in regression model parameter estimates within transition periods. Legend: Data sources are physician billing claims and hospital records; A transition period was defined as $\pm 2$ years around the transition year of 1979 for the transition from ICDA-8 to ICD-9-CM and around the transition year of 2005 for the transition from ICD-9-CM to ICD-10-CA; Horizontal dashed line represents the upper control limit of 19.8. Vertical solid lines represent transition years (1979 and 2005); Vertical dashed lines represent the beginning and end of the transition periods (1977 – 1981 and 2003—2007)

Figure 2 displays the chronic health conditions with no out-of-control observations in any of the transition periods: acute myocardial infarction, acute cerebrovascular disease, colon cancer, and lung and respiratory cancers. Their respective maximum Hotelling’s T^2^ statistics were 16.0, 19.5, 17.2, and 17.3 during the first transition period and 10.4, 12.8, 9.5, and 13.7 during the second transition period; these values are lower than the UCL (Table 4).

In the sensitivity analysis, in which a transition period was defined as $\pm 1$ year around a transition year, the results were similar to the results for the main analysis when a transition period was defined as $\pm 2$ year around a transition year. The exceptions were for osteoarthritis, asthma, and breast cancer. These health conditions had no out-of-control observations within the transition periods (see Figure S2, Figure S3, and Table S1, Additional file 1).

We conducted separate analyses for physician billing claims and hospital records. For the former, there were 10 chronic health conditions (i.e., mood and anxiety disorders, hypertension, anemia, diabetes, asthma, acute cerebrovascular disease, cataracts, breast cancer, prostate cancer, and skin cancer) with significant changes in regression model parameter estimates within the transition period from ICDA-8 to ICD-9-CM (see Figure S4 and Figure S5, Additional file 1). For hospital records, there were six chronic health conditions (i.e., menstrual disorders, acute cerebrovascular disease, breast cancer, colon cancer, lung and respiratory cancers, and skin cancer) with significant changes in regression model parameter estimates within the first transition period and one chronic health condition (i.e., skin cancer) with a significant change in regression model parameter estimates within the second transition period (i.e., when transitioning from ICD-9-CM to ICD-10-CA; see Figure S6 and Figure S7, Additional file 1).

## Discussion

We used control charts to monitor the estimated regression model parameters for 16 chronic health conditions during a 45-year period (1974–2018) when three different ICD versions were used to record diagnoses in administrative health data. We focused on the effect of changes in the ICD version on the prevalence of chronic health conditions, which can result in real changes in the usage, meaning, and interpretation of diagnosis codes [7].

Our results showed that the estimated regression model parameters for most of the investigated chronic health conditions changed significantly during the transition from ICDA-8 to ICD-9-CM. There was no significant changes in the estimated regression model parameters during the transition from ICD-9-CM to ICD-10-CA when both data sources (i.e., physician billing claims and hospital records) were combined. Similar results were obtained when we examined trends in physician billing claims and hospital records separately. However, the exception to this was that we did detect a significant change in the estimated regression model parameters for skin cancer during the transition from ICD-9-CM to ICD-10-CA in hospital records.

These findings may also have occurred because there may have been less standardized training in diagnosis coding methodologies in the late 1970s than in the 2000s, and also less opportunity to communicate amongst healthcare coders, particularly in rural and remote areas of the province of Manitoba, to share information about coding practices.

Databases that include more than one ICD version represent a challenge for trend analyses because significant change in the prevalence estimates of chronic health conditions could emerge solely from the change in coding version, independent of true change in population health [43]. In other to mitigate the effect of change in ICD version on trend analysis, Janssen and Kunst [44] examined five ICD revisions in six European countries and recommended aggregating ICD codes into broader, clinically meaningful groups to reduce the impact of discontinuities in individual codes on trend estimates.

One of the strengths of this study is the population-based data that were used in trend estimation, which ensure generalizability of the results across the entire population of this Canadian province. Also, this study has a long time span, which captured data coded using three ICD versions. These strengths provide an opportunity for longitudinal research covering multiple ICD versions and a unique ability to examine changes within these ICD versions. The inclusion of ICDA-8 in this study aids in filling a knowledge gap; previous studies have focused on the transition from ICD-9 to ICD-10 only [45–48]. Tracking chronic health conditions across multiple decades can contribute to answering questions related to generational impacts of chronic health conditions [49, 50]. We considered 16 chronic health conditions that vary in prevalence and encompass multiple body systems, unlike previous studies that only focused on a single health condition or a single category of health conditions [51–53]. Finally, our methodology can be applied to other health conditions and to data from other Canadian provinces/territories, as well as to data from international jurisdictions.

This study is not without its limitations. Transitions between ICD versions may not be the only factor responsible for the significant changes in prevalence estimates. Prevalence of chronic health conditions derived from administrative health data may be influenced by changes in healthcare providers, including the number of primary care providers, the number and types of specialists, and the availability of other types of care (e.g., emergency departments, long-term care) [54–57]. Also, we limited our attention to modelling trends in prevalence and not trends in incidence of the selected chronic health conditions; different results might have arisen if we had focused on incidence. Furthermore, an out-of-control signal in our control charts analysis indicates a significant change in at least one of the regression model parameters; the specific coefficient(s) responsible for the signal is (are) not directly identified. However, this is not disadvantageous to our study since we were interested in detecting out-of-control signal(s), not the specific parameter responsible for signal detection.

Administrative health data have a number of strengths for research about chronic health conditions; they are relatively inexpensive to access and process and many data repositories now capture multiple decades of data [58, 59]. However, when integrating chronic disease information that arises across different versions of the ICD, there is the potential for misinterpretation of changes in trends if changes in ICD version are not accounted for. Changes in ICD versions captured in administrative health data require crosswalks of the diagnoses if data sources are to be integrated. Crosswalks between three ICD versions (ICDA-8, ICD-9-CM, and ICD-10-CA) for multiple chronic health conditions were recently developed [7].

## Conclusion

In conclusion, we observed that the prevalence estimates of most of the investigated chronic health conditions were significantly affected when transitioning from ICDA-8 to ICD-9-CM, but not when transitioning from ICD-9-CM to ICD-10-CA. The findings of this study will benefit researchers and public health decision makers that rely on administrative health data spanning multiple decades to estimate change in chronic health condition prevalence.

## Supplementary Information

**Additional file 1:** U-Statistic Definition. **Table S1.** Summary of Hotelling’s T^2^ statistics for chronic health conditions in the transition periods. **Figure S1.** Goodness-of-fit statistics for negative binomial and Poisson regression models for 16 chronic health conditions. **Figure S2.** Chronic health conditions with significant changes in regression coefficients within the transition periods. **Figure S3.** Chronic health conditions with no significant changes in regression coefficients within the transition periods. **Figure S4.** Chronic health conditions with significant changes in regression model parameter estimates, physician billing claims. **Figure S5.** Chronic health conditions with no significant changes in regression model parameter estimates, physician billing claims. **Figure S6.** Chronic health conditions with significant changes in regression model parameter estimates, hospital records. **Figure S7.** Chronic health conditions with no significant changes in regression model parameter estimates, hospital records.

## Data Availability

The data that support the findings of this study are not publicly available due to Manitoba privacy restrictions. These data are available with submission of appropriate ethics approval forms to the Health Research Ethics Board of the University of Manitoba (see https://www.umanitoba.ca/research/orec/ethics_medicine/forms.html for more details), and data access approval forms to the Manitoba Health Information Privacy Committee (see https://www.gov.mb.ca/health/hipc/submission.html for more details).
