# Supplementary material for: Transitions between versions of the International Classification of Diseases and chronic disease prevalence estimates from administrative health data: a population-based study
Source: BMC Public Health. 2022 Apr 9;22:701. doi: 10.1186/s12889-022-13118-8 (PMC8994899; doi:10.1186/s12889-022-13118-8)
Supplement: Supplementary file 1 — Additional file 1: U-Statistic Definition. Table S1. Summary of Hotelling’s T2 statistics for chronic health conditions in the transition periods. Figure S1. Goodness-of-fit statistics for negative binomial and Poisson regression models for 16 chronic health conditions. Figure S2. Chronic health conditions with significant changes in regression coefficients within the transition periods. Figure S3. Chronic health conditions with no significant changes in regression coefficients within the transition periods. Figure S4. Chronic health conditions with significant changes in regression model parameter estimates, physician billing claims. Figure S5. Chronic health conditions with no significant changes in regression model parameter estimates, physician billing claims. Figure S6. Chronic health conditions with significant changes in regression model parameter estimates, hospital records. Figure S7. Chronic health conditions with no significant changes in regression model parameter estimates, hospital records. [file 12889_2022_13118_MOESM1_ESM.docx]

**Additional File 1**

**U-Statistic Definition**

The coefficients for the negative binomial regression model were estimated by $\hat{\boldsymbol{\beta}_{j}}\boldsymbol{=}\left( b_{kj} \right)$ for $k=0, 1, \ldots,(p-1)$, where$p$ is the number of estimated parameters, using maximum likelihood estimation. The estimated parameters were adjusted for autocorrelation using a U-statistic [1] defined as

$\mathbf{U}_{j}=\left( {b'}_{0j},{b'}_{1j},\ldots,{b'}_{pj} \right)^{'}=\left\{ \begin{aligned} {\hat{\mathbf{A}}}_{1}, \mathrm{when} j=1 \\ {\hat{\mathbf{A}}}_{j}\boldsymbol{-}\boldsymbol{\Sigma}_{j,j-1}\boldsymbol{\Sigma}_{j-1,j-1}^{-1}{\hat{\mathbf{A}}}_{j-1}, \mathrm{when} j=2,\ldots,m \end{aligned} \right.$ (1)

where ${b'}_{0j}$ is the adjusted estimated intercept and ${b'}_{1j},\ldots,{b'}_{pj}$ are the adjusted estimated slope parameter for the $j^{th}$ year, ${\hat{\mathbf{A}}}_{j}={{\hat{\boldsymbol{\beta}}}_{j}}/\left( 1-\varphi\right)$, $\varphi$ is the estimated autocorrelation ﻿coefficient computed with the Durbin-Watson test statistic, and $\boldsymbol{\Sigma}_{(.,.)}$ is the covariance matrix of ${\hat{\mathbf{A}}}_{j}$ [1]. The $\mathbf{U}_{\boldsymbol{j}}s$ are assumed to be independent and normally distributed with mean vector and covariance matrix respectively expressed as

$\boldsymbol{\mu}_{\boldsymbol{U}_{\boldsymbol{j}}}=\left\{ \begin{aligned} {\hat{\mathbf{A}}}_{1}, \mathrm{when} j=1 \\ \boldsymbol{\mu}_{{\hat{\boldsymbol{A}}}_{j}}\boldsymbol{-}\boldsymbol{\Sigma}_{j,j-1}\boldsymbol{\Sigma}_{j-1,j-1}^{-1}\boldsymbol{\mu}_{{\hat{\boldsymbol{A}}}_{j}-1}, \mathrm{when} j=2,\ldots,m \end{aligned} \right.$ (2)

$\boldsymbol{\Sigma}_{\boldsymbol{U}_{\boldsymbol{j}}}=\left\{ \begin{aligned} \boldsymbol{\Sigma}_{1,1}\boldsymbol{,} \mathrm{when} j=1 \\ \boldsymbol{\Sigma}_{j,j}\boldsymbol{-}\boldsymbol{\Sigma}_{j,j-1}\boldsymbol{\Sigma}_{j-1,j-1}^{-1}\boldsymbol{\Sigma}_{j-1,j}, \mathrm{when} j=2,\ldots,m \end{aligned} \right.$ (3)

**Supplementary Results**

Table S1: Summary of Hotelling’s T^2^ statistics for chronic health conditions in the transition periods

| **ID** | **Chronic Health Condition** | **Transition period from ICDA-8 to ICD-9-CM** | | **Transition period from ICD-9-CM to ICD-10-CA** | |
| --- | --- | --- | --- | --- | --- |
|  |  | **Max T^2^** | **Average T^2^** | **Max T^2^** | **Average T^2^** |
| 1 | Mood and anxiety disorders | **22.4** | 16.9 | 10.8 | 6.4 |
| 2 | Menstrual disorders | **21.4** | 16.6 | 9.7 | 7.9 |
| 3 | Hypertension | **20.9** | 13.1 | 8.1 | 6.5 |
| 4 | Osteoarthritis | 11.9 | 9.9 | 12.3 | 8.9 |
| 5 | Anemia | **28.4** | 17.2 | 9.7 | 8.2 |
| 6 | Diabetes | **21.7** | 15.0 | 9.7 | 6.9 |
| 7 | Asthma | 15.2 | 11.3 | 12.8 | 8.5 |
| 8 | Acute myocardial infarction | 16.0 | 10.9 | 10.4 | 9.1 |
| 9 | Heart valve disorders | **23.0** | 16.2 | 6.6 | 5.9 |
| 10 | Acute cerebrovascular disease | 19.5 | 13.8 | 12.8 | 7.8 |
| 11 | Cataracts | **21.4** | 14.3 | 10.5 | 9.2 |
| 12 | Breast cancer | 11.1 | 10.7 | 8.8 | 6.8 |
| 13 | Colon cancer | 16.5 | 11.2 | 9.5 | 8.4 |
| 14 | Lung & respiratory cancers | 12.9 | 10.9 | 13.7 | 12.2 |
| 15 | Prostate cancer | **29.9** | 19.5 | 10.5 | 6.0 |
| 16 | Skin cancer | **23.1** | 20.6 | 9.2 | 5.9 |

Data sources are physician billing claims and hospital records; Boldface font indicates a test statistic value greater than the upper control limit of 19.8; The transition period was defined as$\pm1$ year around the transition year of 1979 for the transition from ICDA-8 to ICD-9-CM, and around the transition year of 2005 for the transition from ICD-9-CM to ICD-10-CA.


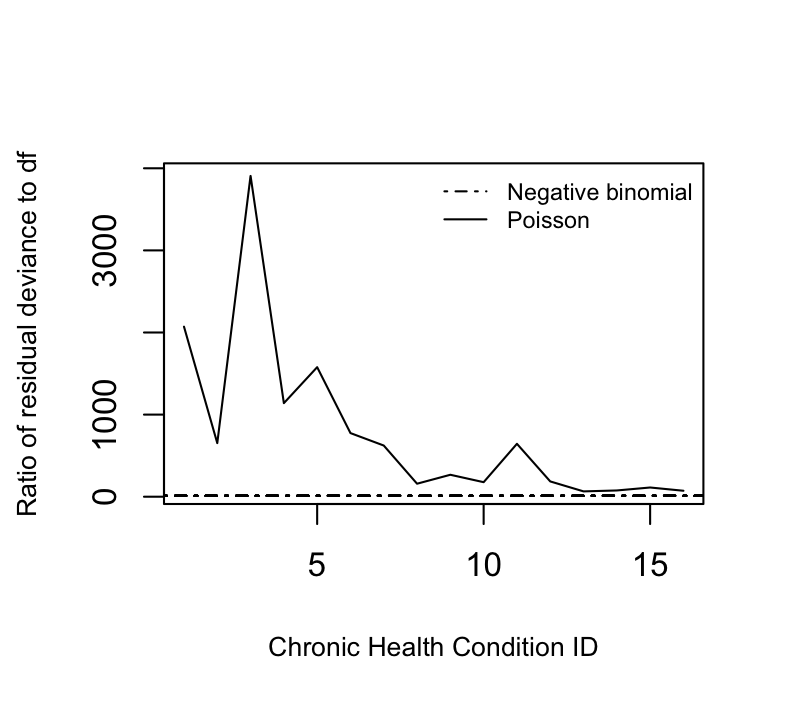


Figure S1: Goodness-of-fit statistics for negative binomial and Poisson regression models for 16 chronic health conditions

Legend: df = degrees of freedom.


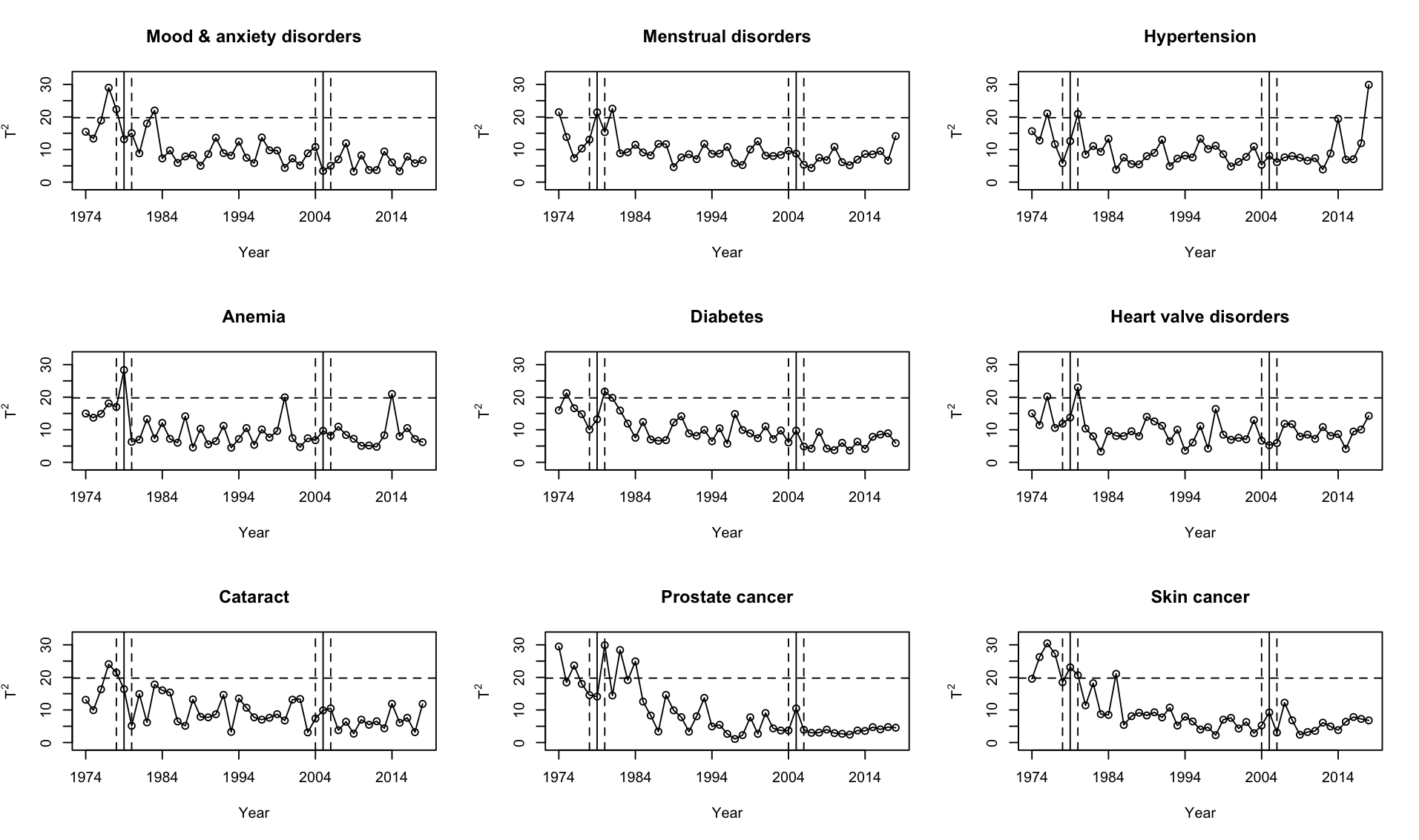
Figure S2: Chronic health conditions with significant changes in regression model parameter estimates within transition periods

Legend: Data sources are physician billing claims and hospital records; A transition period was defined as$\pm1$ year around the transition year of 1979 for the transition from ICDA-8 to ICD-9-CM and around the transition year of 2005 for the transition from ICD-9-CM to ICD-10-CA; Horizontal dashed line represents the upper control limit of 19.8; Vertical solid lines represent transition years (1979 and 2005). Vertical dashed lines represent the beginning and end of the transition periods (1978 - 1980 and 2004 - 2006).


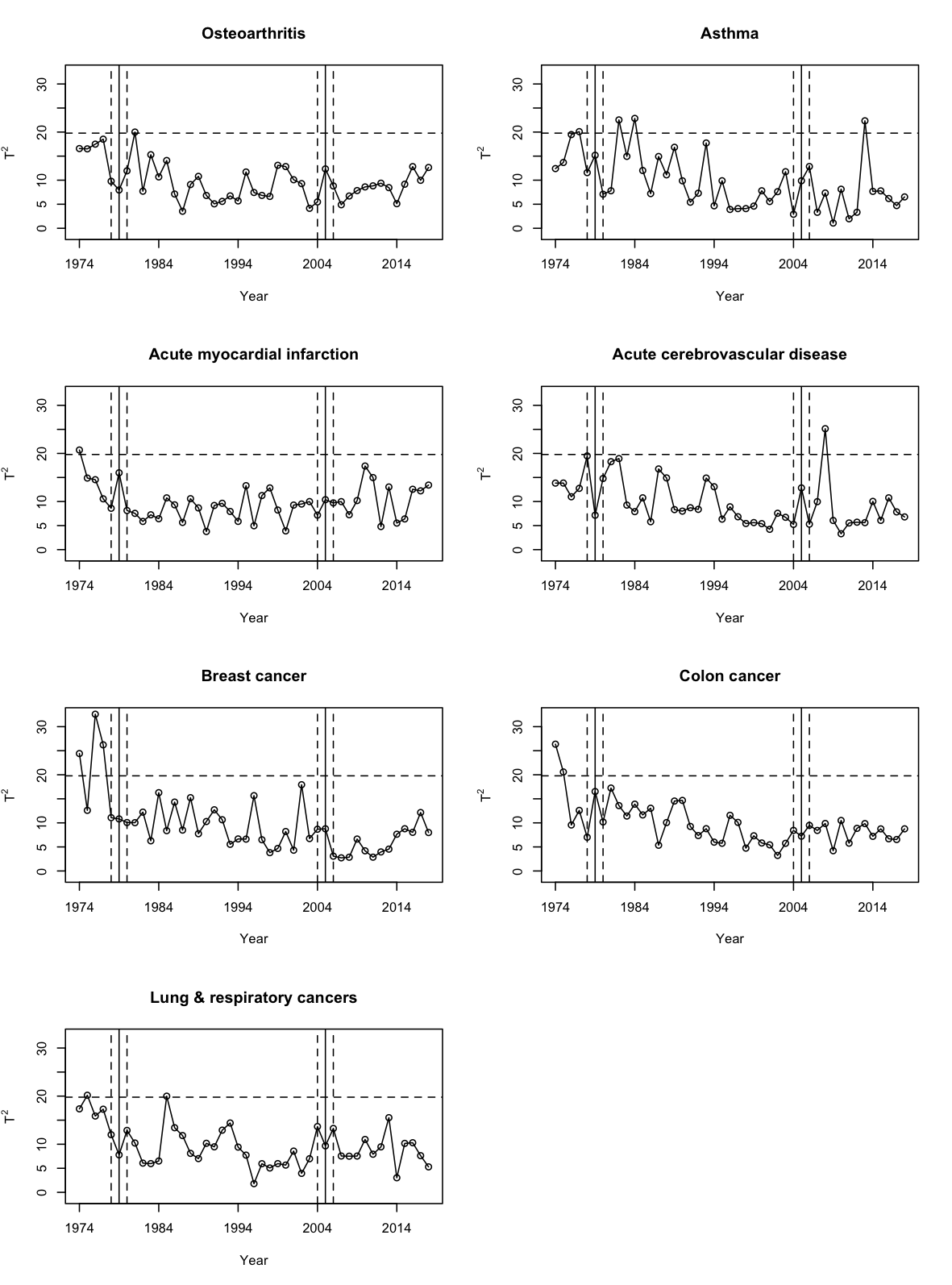
Figure S3: Chronic health conditions with no significant changes in regression model parameter estimates within transition periods

Legend: Data sources are physician billing claims and hospital records; A transition period was defined as$\pm1$ year around the transition year of 1979 for the transition from ICDA-8 to ICD-9-CM and around the transition year of 2005 for the transition from ICD-9-CM to ICD-10-CA; Horizontal dashed line represents the upper control limit of 19.8; Vertical solid lines represent transition years (1979 and 2005); Vertical dashed lines represent the beginning and end of the transition periods (1978 – 1980 and 2004 – 2006).


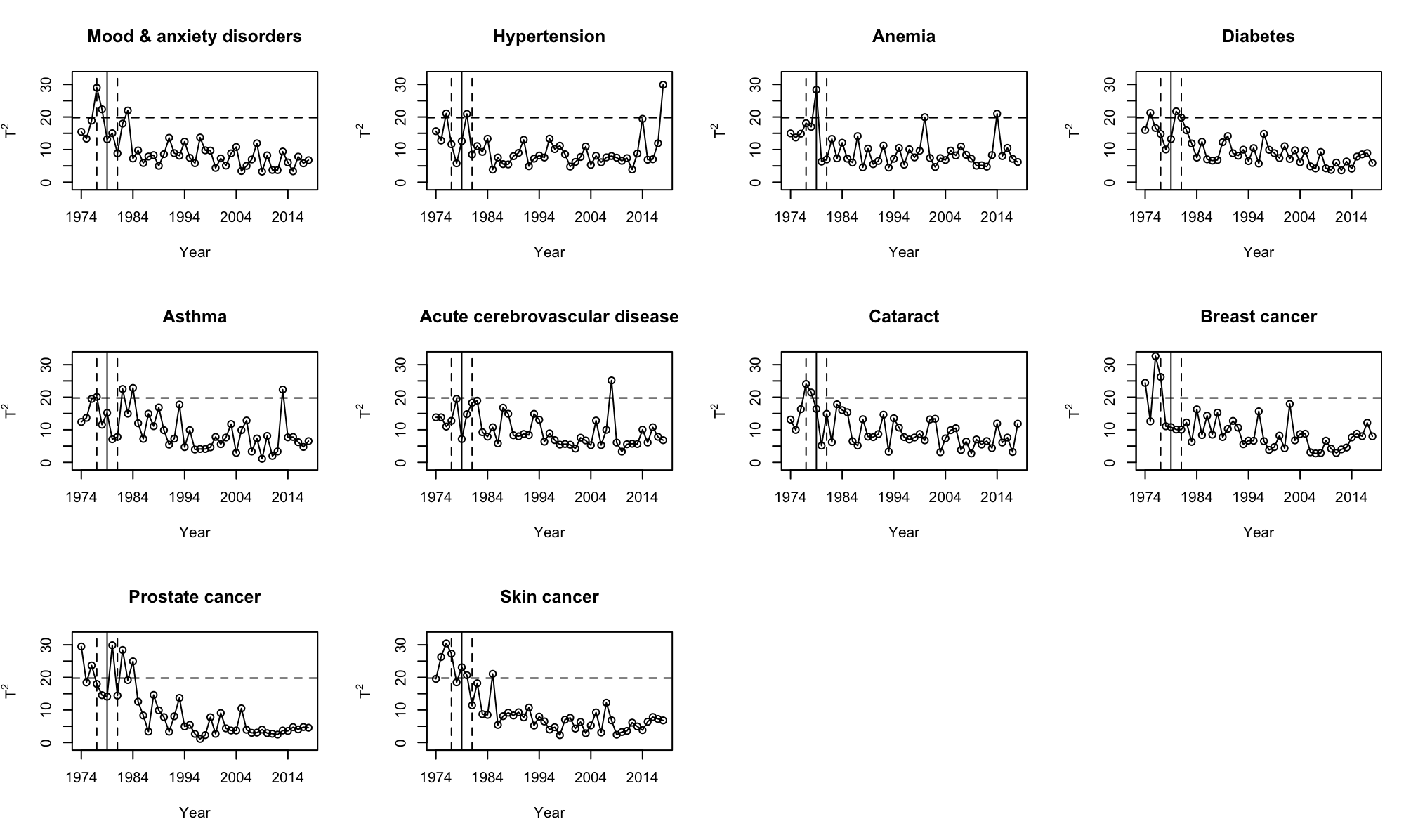
Figure S4: Chronic health conditions with significant changes in regression model parameter estimates, physician billing claims

Legend: A transition period was defined as$\pm2$ years around the transition year of 1979 for the transition from ICDA-8 to ICD-9-CM; Horizontal dashed line represents the upper control limit of 19.8; Vertical solid line represent transition year (1979); Vertical dashed lines represent the beginning and end of the transition period (1977 – 1981).


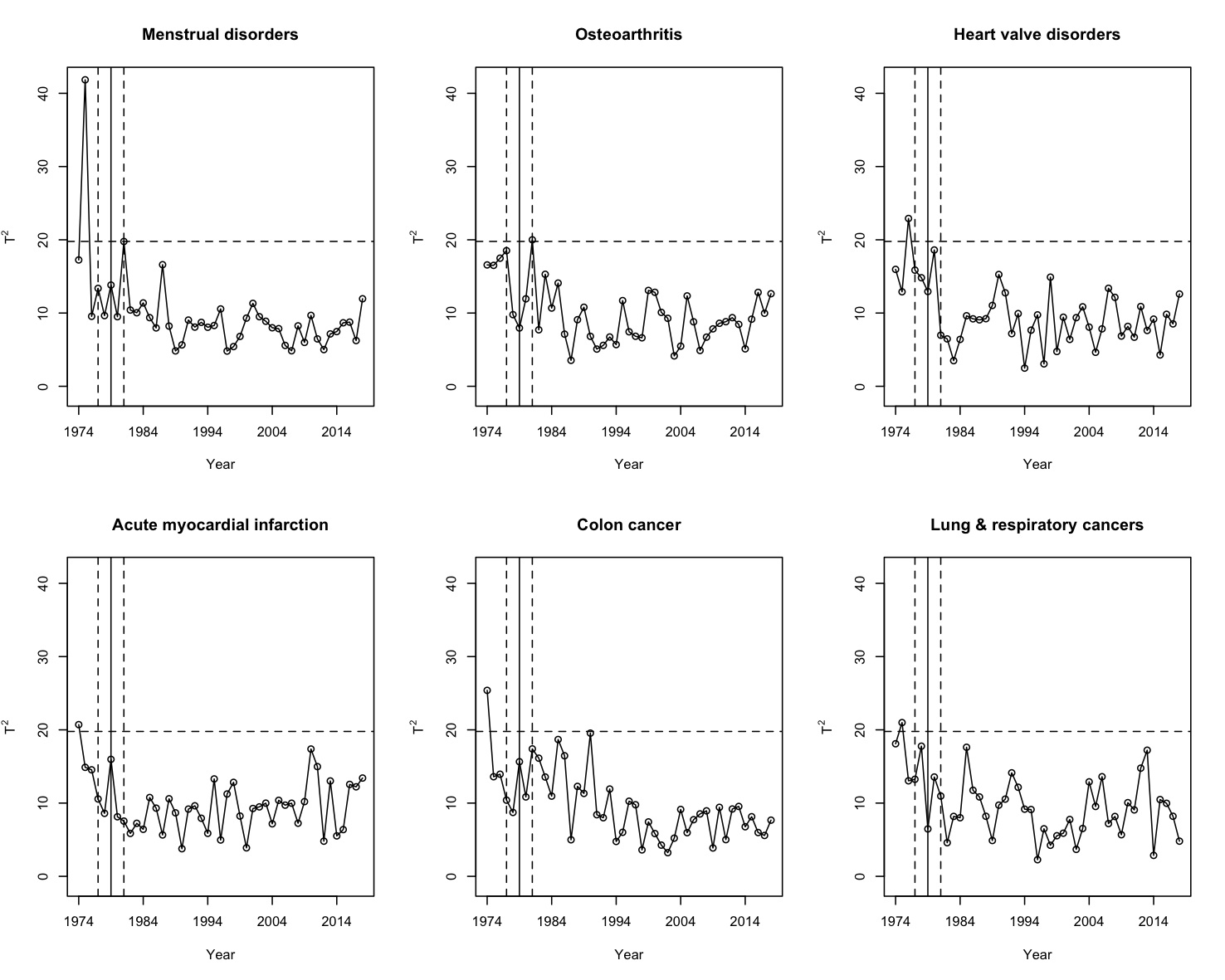


Figure S5: Chronic health conditions with no significant changes in regression model parameter estimates, physician billing claims

Legend: A transition period was defined as$\pm2$ years around the transition year of 1979 for the transition from ICDA-8 to ICD-9-CM; Horizontal dashed line represents the upper control limit of 19.8; Vertical solid line represent transition year (1979); Vertical dashed lines represent the beginning and end of the transition period (1977 – 1981).


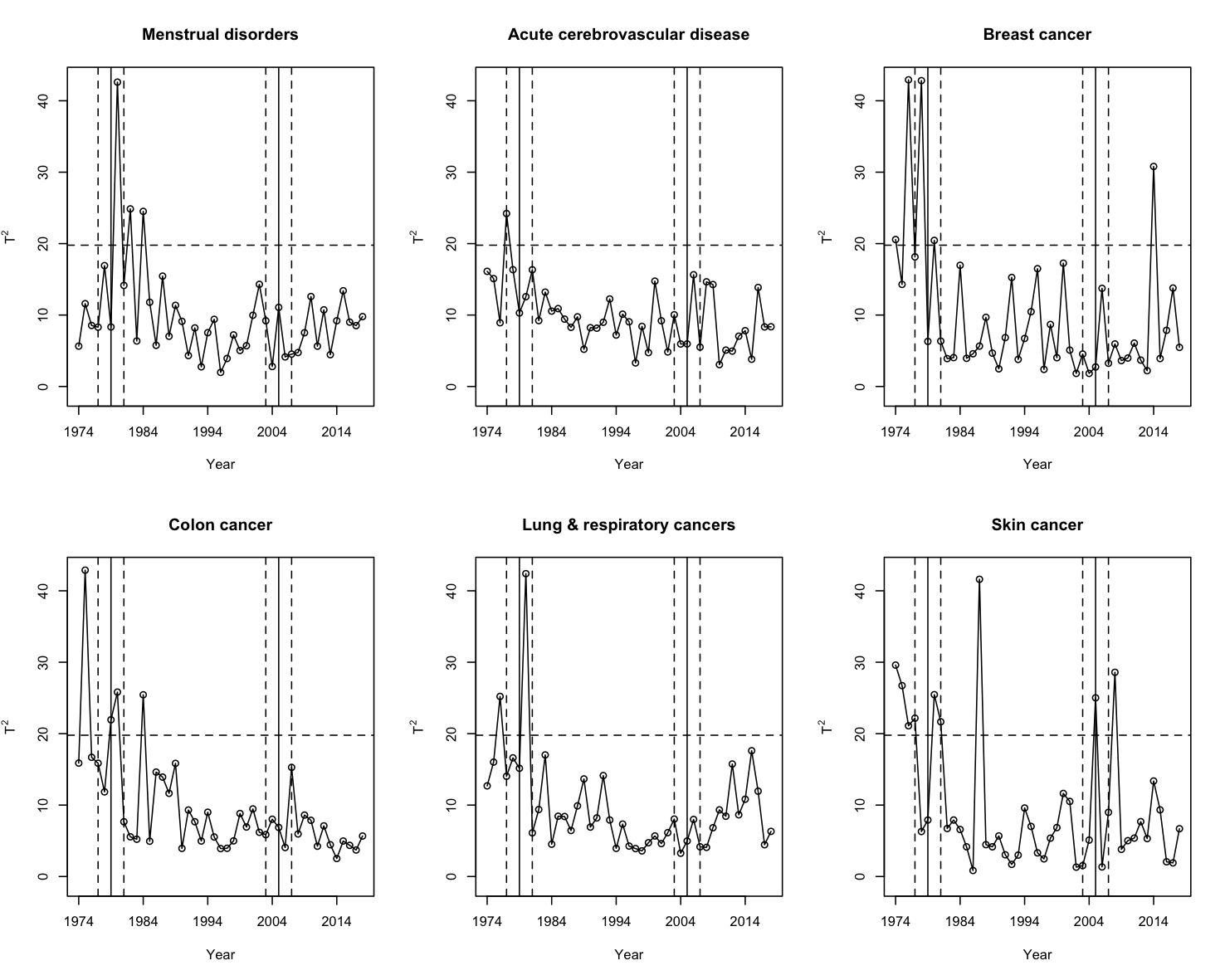


Figure S6: Chronic health conditions with significant changes in regression model parameter estimates, hospital records

Legend: A transition period was defined as$\pm2$ years around the transition year of 1979 for the transition from ICDA-8 to ICD-9-CM and around the transition year of 2005 for the transition from ICD-9-CM to ICD-10-CA; Horizontal dashed line represents the upper control limit of 19.8; Vertical solid lines represent transition years (1979 and 2005). Vertical dashed lines represent the beginning and end of the transition periods (1977 - 1981 and 2003 - 2007).


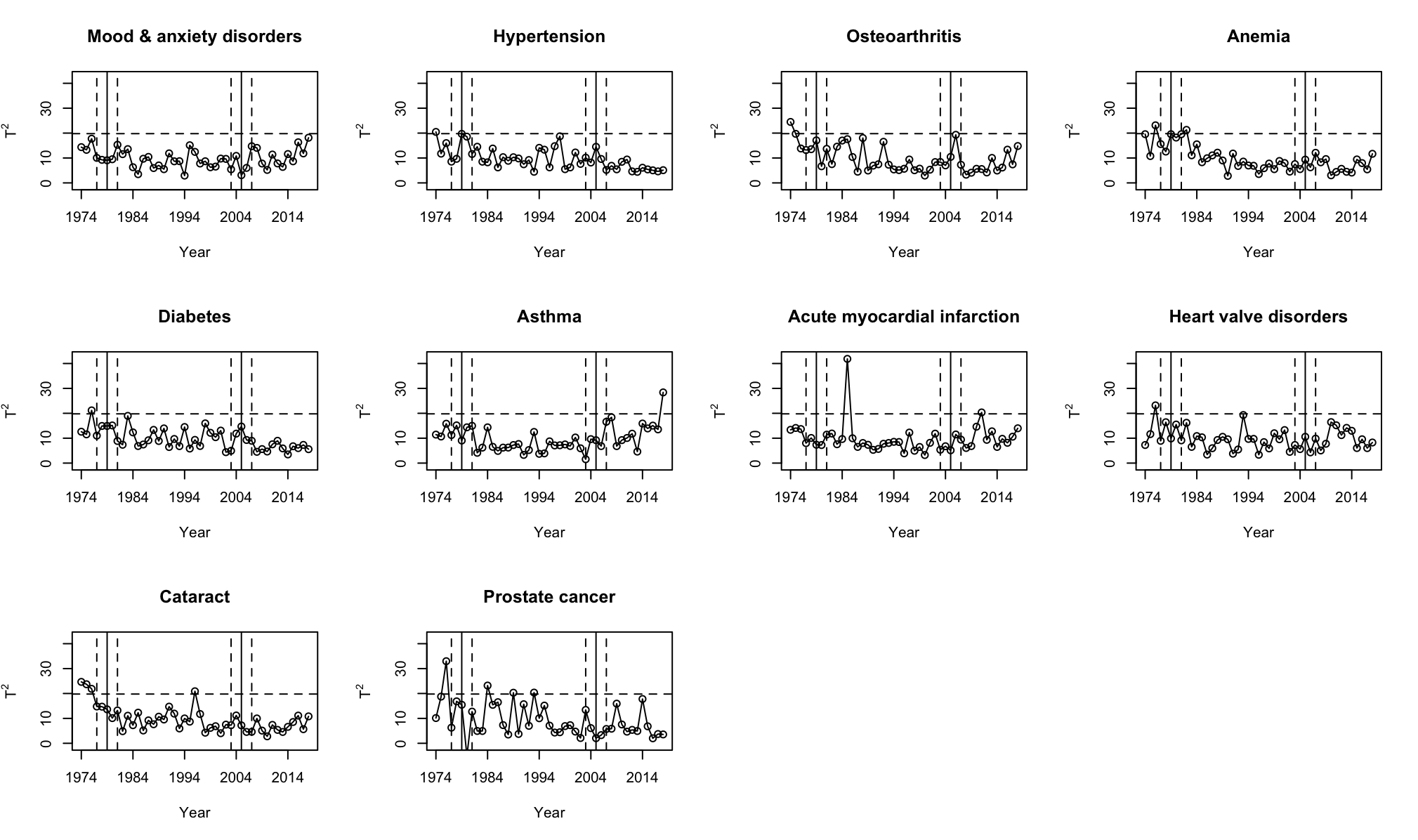
Figure S7: Chronic health conditions with no significant changes in regression model parameter estimates, hospital records

Legend: A transition period was defined as$\pm2$ years around the transition year of 1979 for the transition from ICDA-8 to ICD-9-CM and around the transition year of 2005 for the transition from ICD-9-CM to ICD-10-CA; Horizontal dashed line represents the upper control limit of 19.8; Vertical solid lines represent transition years (1979 and 2005); Vertical dashed lines represent the beginning and end of the transition periods (1977 – 1981 and 2003 - 2007).

**Reference**

1. Khedmati M, Niaki STA. Phase II monitoring of general linear profiles in the presence of between-profile autocorrelation. Qual Reliab Eng Int. 2016;32:443–52. doi:10.1002/qre.1762.
